# Coexistence of early microinvasive endometrioid adenocarcinoma and CIN3 in the uterine cervix in a 32-year-old Japanese woman

**DOI:** 10.1186/1746-1596-6-51

**Published:** 2011-06-10

**Authors:** Tadashi Terada

**Affiliations:** 1 Department of Pathology, Shizuoka City Shimizu Hospital, Shizuoka, Japan; 2 Department of Pathology, Shizuoka City Shimizu Hospital, Miyakami 1231 Shimizu-Ku, Shizuoka 424-8636, Japan

**Keywords:** early microinvasive adenocarcinoma, CIN3, uterine cervix, histopathology, immunohistochemistry

## Abstract

Simultaneous occurrence of early microinvasive endometrioid adenocarcinoma (EMEA) and CIN 3 in the uterine cervix is very rare in Japan. A 32-year-old Japanese woman was pointed out to have atypical cells in the cervical cytology. Colposcopic examination revealed irregular lesions in the cervix, and a biopsy showed simultaneous EMEA and CIN3. The EMEA was grade I and CIN3 corresponded to severe dysplasia/carcinoma in situ. Hysterectomy and lymph nodes dissection were performed. Grossly, mucosal irregularity and erosion were seen in the cervix. No tumor formation was recognized. The cervix was examined by serial sections. Microscopically, there were a tiny adenocarcinoma (0.5 cm in diameter and 0.3 cm in depth) and broad areas of CIN3. The adenocarcinoma was EMEA without mucins. The EMEA was FIGO stage 1A1. Immunohistochemically, the EMEA was positive for pancytokeratins (AE1/2 +++, CAM5.2 ++), cytokeratin (CK) 34βE12 +, CK5/6 +, CK7 +, CK18 +++, CK19 ++, CA19-9 +, CA125 +++, p53 +, ER +++, PgR +++, while it was negative for CK8, CK14, CK20, EMA, vimentin, CEA, desmin, smooth muscle actin, p63, chromogranin, synaptophysin, CD56, CD68, HER2/neu, MUC1, MUC2, MUC5AC, and MUC6. The CIN 3 was positive for pancytokeratins (AE1/2 +++, CAM5.2 +), cytokeratin (CK) 34βE12 +++, CK5/6 +++, CK7 +, EMA, CA19-9 +, CA125 +, p53 +, p63 +++, ER +++, and MUC1 +, while it was negative for CK8, CK14, CK18, CK19, CK20, vimentin, CEA, desmin, smooth muscle actin, chromogranin, synaptophysin, CD56, CD68, PgR, HER2/neu, MUC2, MUC5AC and MUC6. The lymph nodes showed no metastatic lesions (0/34). In conclusion, the author reported a rare case of simultaneous EMEA and CIN 3 with extensive immunohistochemical findings.

## Introduction

Malignant neoplasms of the uterine cervix are frequent in Japan. Most of them are squamous cell carcinoma and cervical intraepithelial neoplasm (CIN), and adenocarcinoma and its precursor lesions are rare in Japan.

Adenocarcinoma of the uterine cervix was classified into adenocarcinoma NOS, mucinous adenocarcinoma, endometrioid adenocarcinoma, clear cell adenocarcinoma, serous adenocarcinoma mesonephric adenocarcinoma, early microinvasive adenocarcinoma, and adenocarcinoma in situ [1]. The mucinous adenocarcinoma was subclassified into endocervical, intestinal, signet-ring cell, minimal deviation, and villoglandular subtypes [1]. Glandular dysplasia equivalent to squamous dysplasia is also present.

More than 90% of uterine tumorous lesions are squamous cell carcinoma and its precursor lesions. The precursor lesions of the squamous cell carcinoma had traditionally been called dysplasia (mild, moderate, and severe) and carcinoma in situ (CIS). Recently, they have been termed as cervical intraepithelial neoplasm (CIN) or squamous intraepithelial lesions (SIL) [1]. CIN is categorized as CIN 1-3, and SIL as low grade SIL (CIN 1) and high grade SIL(CIN2-3/CIS) (HGSIL) [1]. These lesions are known to be frequently associated with sexual intercourses and human papilloma virus (HPV) infection [1].

According to WHO [1], adenocarcinoma is associated with CIN in about 40% of cases. However, Brown et al [2] reported that only 1 case of adenocarcinoma was found in 105 cases of CIN 3. Therefore, the coexistence of adenocarcinoma and CIN 3 is very rare. The author herein reports a case of simultaneous early microinvasive endometrioid adenocarcinomna (EMEC) and CIN 3 in a young Japanese woman.

### Case report

**Figure 1:**
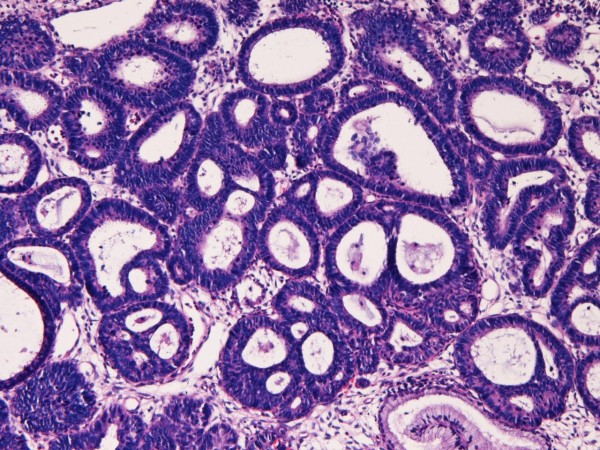
**Biopsy of uterine cervix**. The biopsy shows atypical glands with hyperchromatic nuclei and with structural atypia. This lesion is regarded as adenocarcinoma. HE, ×100.

**Figure 2:**
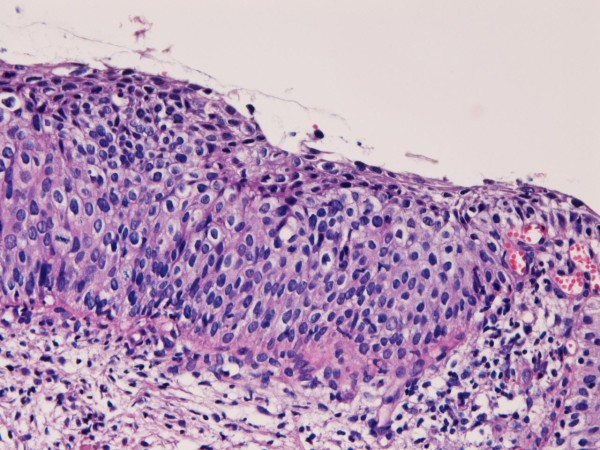
**Biopsy of uterine cervix**. The biopsy shows atypical squamous lesion. The atypical cells showed hyperchromatic nuclei and occupy all layers. The polarity and stratification are lost. The lesion was regarded as CIN3. HE, ×100.

A 32-year-old Japanese woman was found to have atypical cells in the cervical cytology at routine check. Colposcopic examination revealed irregular lesions in the cervix. A biopsy revealed simultaneous adenocarcinoma (Figure 1) and CIN3 (Figure 2). The adenocarcinoma have grade I atypia and CIN3 corresponded to severe dysplasia/carcinoma in situ.

**Figure 3:**
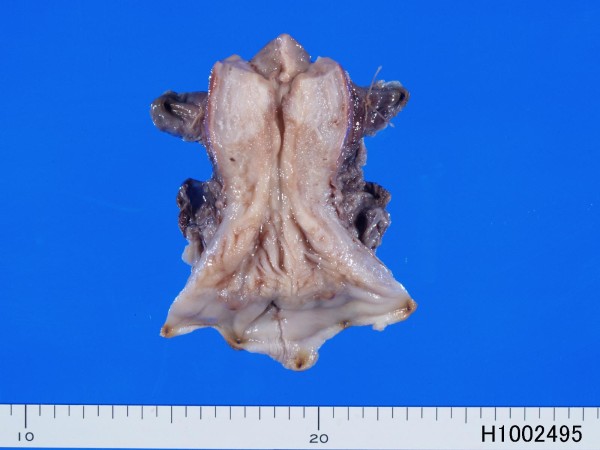
**Gross features of resected uterus**. The cervix shows irregularity and erosions. No apparent tumor is seen.

**Figure 4:**
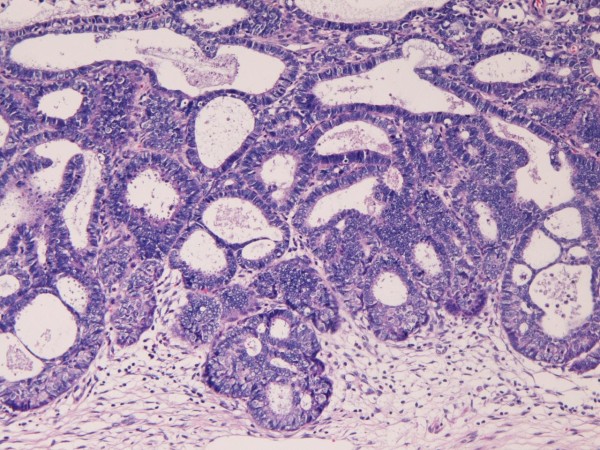
**Microscopic features of the resected uterus**. The adenocarcinoma component The adenocarcinoma is endometrioid adenocarcinoma. The carcinoma is small with mild invasion. HE ×50.

**Figure 5:**
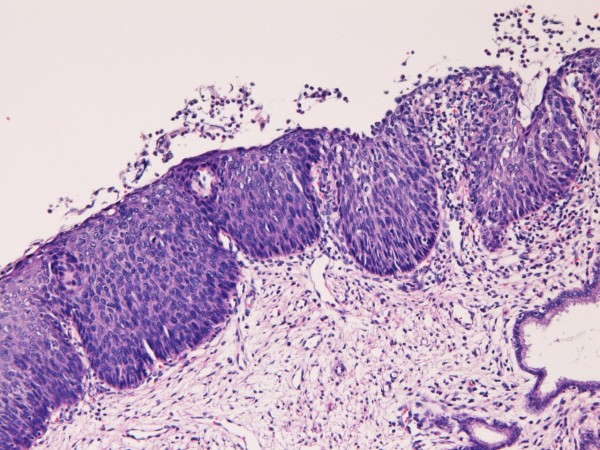
**Microscopic features of the resected uterus**. The CIN3 component. CIN3 without invasion is seen. HE ×50.

Hysterectomy and lymph nodes dissection were performed. Grossly, mucosal irregularity and erosion were seen in the cervix (Figure 3). No tumor formation was recognized. The cervix was examined by serial sections. There were a tiny adenocarcinoma (0.5 cm in diameter and 0.3 cm in depth) (Figure 4) and broad areas of CIN3 (Figure 5). Both lesions are separate, and no merges between both lesions were seen. The adenocarcinoma was EMEA without mucins, as revealed by negative alcian blue/PAS staining. The lymph nodes were negative for metastasis (0/34). No metastatic foci were found in the body. The EMEA was FIGO stage 1A1.

**Figure 6:**
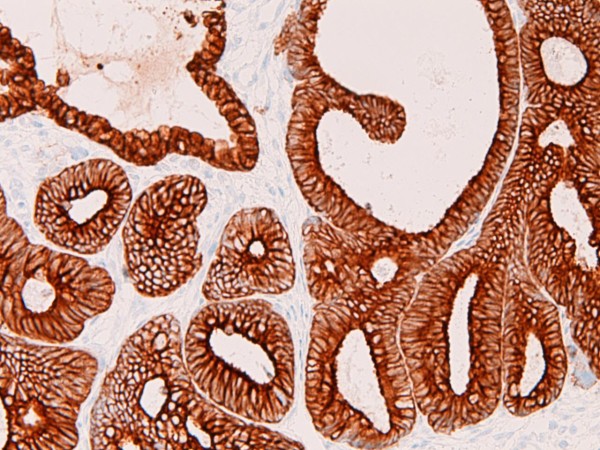
**Immunohistochemical features**. The adenocarcinoma component is strongly positive for cytokeratin 18. ×100.

**Figure 7:**
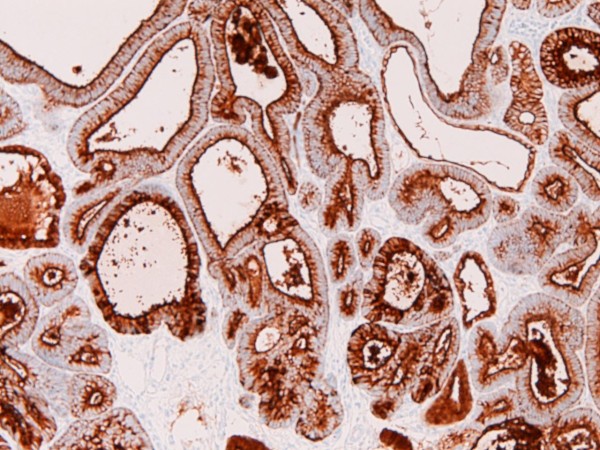
**Immunohistochemical features**. The adenocarcinoma component is strongly positive for CA125. ×100.

**Figure 8:**
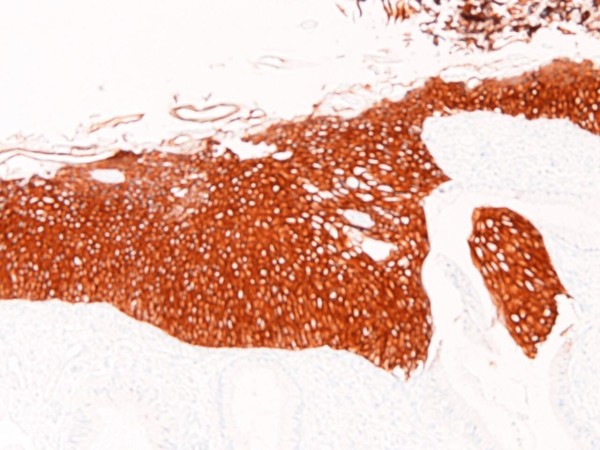
**Immunohistochemical features**. The CIN3 component was strongly positive for cytokeratin 34βE12. ×100.

**Figure 9:**
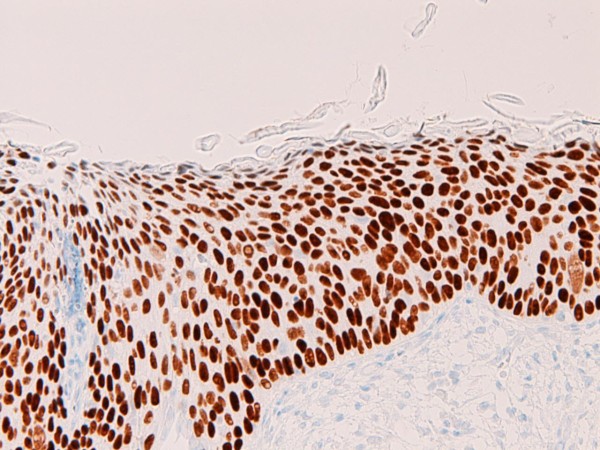
**Immunohistochemical features**. The CIN3 component was strongly positive for p63. ×100.

**Figure 10:**
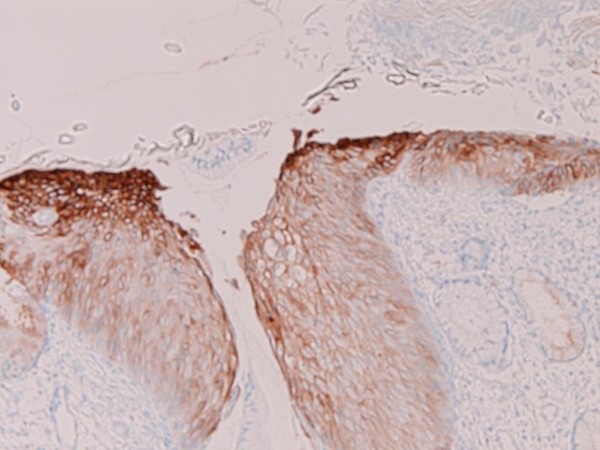
**Immunohistochemical features**. The CIN3 component was positive for MUC1. ×100.

An immunohistochemical study was performed with the use of DAKO Envision method (Dako Corp., Glostrup, Denmark), as described previously [3-7]. The immunohistochemical results are shown in Table 1. Immunohistochemically, the EMEA was positive for pancytokeratins (AE1/2 +++, CAM5.2 ++), cytokeratin (CK) 34βE12 +, CK5/6 +, CK7 +, CK18 +++(Figure 6), CK19 ++, CA19-9 +, CA125 +++ (Figure 7), p53 +, ER +++, PgR +++, while it was negative for CK8, CK14, CK20, EMA, vimentin, CEA, desmin, smooth muscle actin, p63, chromogranin, synaptophysin, CD56, CD68, HER2/neu, MUC1, MUC2, MUC5AC, and MUC6. The CIN 3 was positive for pancytokeratins (AE1/2 +++, CAM5.2 +), cytokeratin (CK) 34βE12 +++ (Figure 8), CK5/6 +++, CK7 +, EMA, CA19-9 +, CA125 +, p53 +, p63 +++ (Figure 9), ER +++, and MUC1 + (Figure 10), while it was negative for CK8, CK14, CK18, CK19, CK20, vimentin, CEA, desmin, smooth muscle actin, chromogranin, synaptophysin, CD56, CD68, PgR, HER2/neu, MUC2, MUC5AC, and MUC6. The patient is now free from tumor 4 months after the operation.

**Table 1:** Immunohistochemical reagents and results

			Results	
Antigens	Antibodies (clone)	Sources	**EMEA**.	CIN3
Pancytokeratin	AE1/3	Dako Corp. Glostrup, Denmark	+++	+++
Pancytokeratin	CAM5.2	Beckton-Dickinson, CA, USA	++	+
HMWCK	34βE12	Dako	+	+++
CK5/6	D5/16	Dako	+	+++
CK7	N1626	Dako	+	+
CK8	DC10	Dako	-	-
CK14	LL002	Novocastra, Newcastle upon type, UK	-	-
CK 18	DC10	Dako	+++	-
CK 19	RCK 108	Progen, Heidelberg, Germany	++	-
CK 20	K20.8	Dako	-	-
EMA	E29	Dako	-	+
Vimentin	Vim 3B4	Dako	-	-
CEA	polyclonal	Dako	-	-
CA19-9	NS19-9	TBF Lab, Tokyo Japan	+	+
CA125	NS125	TFB Lab	+++	+
Desmin	D33	Dako	-	-
ASMA	1A4	Dako	-	-
p53 protein	DO-7	Dako	+	+
p63	4A4	Dako	-	+++
Chromogranin	DAK-A3	Dako	-	-
Synaptophysin	Polyconal	Dako	-	-
CD56	UJ13A	Dako	-	-
CD68	KP-1	Dako	-	-
ER	M7047	Dako	+++	+++
PgR	1A6	Novocastra	+++	-
HER2/neu	CB11	Ventana Japan, Tokyo	-	-
MUC1	Ma695	Novocastra	-	+
MUC2	CCp58	Novocastra	-	-
MUC5AC	CLH2	Novocastra	-	-
MUC6	CLH5	Novocastra	-	-

+++, strongly positive. ++, moderately positive. +, mildly positive. -, negative. EMEA, early microinvasive endometrioid adenocarcinoma. CIN3, cervical intraepithelial neoplasm grade 3. HMWCK, high molecular weight cytokeratin. CK, cytokeratin. EMA, epithelial membrane antigen. CEA, carcinoembryonic antigen. CA19-9, carbohydrate antigen 19-9. CA125, carbohydrate antigen 125. ASMA, α-smooth muscle antigen. ER, estrogen receptor. PgR, progesterone receptor.

## Discussion

The adenocarcinoma in the present case was early microinvasive with width of 0.5 cm and depth of 0.3 cm. Histologically, the adenocarcinoma resembled endometrial endometrioid adenocarcinoma (grade 1), and negative for mucins. Therefore, the adenocarcinoma was EMEA [1]. The diagnosis of CIN 3 is apparent in the present case [1].

Squamous cell carcinoma and adenocarcinoma can coexist in the uterine cervix [8-13]; there are several case reports of this in the English literature [8-13]. From the standpoint of CIN, Brown et al [2] reported that 16 cases of cervical glandular atypia and only 1 case adenocarcinoma in situ were found in 105 cases of CIN 3. From the standpoint of cervical adenocarcinoma, Maier and Norris [9] reported that 99 cases of CIN were detected in 230 cases of cervical adenocarcinoma. About half of such cases were CIN 2 or CIN 3 [9]. There is only one report that there is an association of adenocarcinoma in situ and cervical squamous cell carcinoma [10]. However, cases of simultaneous adenocarcinoma and CIN3 are very rare in the English literature [10,14-19]. In addition, cases of simultaneous early microinvasive adenocarcinoma and CIN3 are very rare [17,18]. Furthermore, cases of simultaneous endometrioid adenocarcinoima and CIN3 are extremely rare. Therefore, the present case of coexistence of EMEA and CIN3 appears extremely rare.

In the present study, the cervical lesion is typical CIN3, HGSIL, or CIS. No invasion was recognized. The histological features fulfill the diagnosis of CIN 3 [1]. The adenocarcinoma element in the present study was of EMEA negative for mucins [1]. In both elements, adequate structural and cytological atypia regarded as malignant was recognized. The positive immunoreaction of p53 protein in the present EMEA and CIN3 strongly suggests that both lesions in the present study are malignant.

There have been few immunohistochemical studies in simultaneous squamous neoplasm and adenocarcinoma in the uterine cervix [10]. The present study extensively examined the immunoprofile of EMEA and CIN3. The EMEA and CIN3 showed different immunoprofile, suggesting that they are different neoplasms. The CK profiling of EMEA showed predominant presence of low molecular weight cytokeratin, while CIN3 high-molecular weight cytokeratin. Since squamous lesions express high-molecular weight CK while adenocarcinoma low-molecular weight cytokeratin [20], the present data indicate the CIN3 is squamous lesions and EMEA is adenocarcinoma. The presence of p63 in CIN3 but not in EMEA indicates that CIN3 was squamous lesion and EMEA is not, since p63 is known to be expressed in squamous lesions but not in adenocarcinoma lesions [21]. Both components were negative for CK14, CK17, and CK20, suggesting that these three CKs are not expressed in either lesion. EMA was positive in CIN3, but negative in EMEA, suggesting that EMA is expressed only in CIN lesions. CA125 was strongly positive in EMA and mildly positive in CIN, suggesting that CA125 is mainly expressed in adenocarcinoma element rather than CIN. CA19-9 was mildly expressed in both EMEA and CIN3, suggesting that both lesions have a small amount of this carbohydrate antigen. p53 was positive in both components, suggesting that p53 gene is mutated in both components and that both components are malignant. Both components were strongly positive for ER, being compatible with the presence of ER of female genital organs. However, PgR was strongly positive for EMEA but negative in CIN3, suggesting the presence of PgR in EMEA, but not in CIN. Interestingly, MUC 1 was present in CIN3 but was absent in EMEA. MUC2, MUC5AC and MUC6 were negative in both components. MUC is mucin core protein gene product so that it was previously anticipated that MUCs are present in EMEA. These results indicate CIN3 may be positive for MUC1 and EMEA was negative for MUCs. Both elements were negative for chromogranin, synaptophysin, and CD56, indicating that both components do not show neuroendocrine features. Both components were negative for vimentin, desmin, and α-smooth mucle actin, indicating that these three mesenchymal antigens were absent in EMEA and CIN3. CEA was negative for both components, suggesting that EMEA may be negative for CEA. CD68 was negative in both components, indicating no histiocytic differentiation. Both components were negative for HER2/neu, suggesting that both components were negative for this receptor.

In summary, the author presented a case of simultaneous EMEA and CIN3 of the uterine cervix in a 32-year-old Japanese woman with an emphasis on immunohistochemical findings.

### Consent

Written informed consent was obtained from the patient for publication of this case report and accompanying images. A copy of the written consent is available for review by the Editor-in-Chief of this journal.

## Competing interests

The author declares that they have no competing interests.
